# Case Report: Identification of a *CRYGD* variant in a family with congenital cataract

**DOI:** 10.3389/fmed.2026.1778174

**Published:** 2026-06-10

**Authors:** Junjie Deng, Jianli Ma, Yixiao Li, Wenjing Wang, Chunli Ma, Mengxue Li, Yaqin Jiang, Han Zhang

**Affiliations:** 1 Department of Ophthalmology, Shandong Provincial Hospital Affiliated to Shandong First Medical University, Jinan, Shandong, China; 2 Department of Ophthalmology, Shandong First Medical University and Shandong Academy of Medical Science, Jinan, Shandong, China; 3 Department of Ophthalmology, Weifang Eye Hospital, National Key Clinical specialty, Zhengda Guangming Eye Group, Weifang, Shandong, China; 4 Weifang Eye Institute, Weifang, Shandong, China; 5 Department of Ophthalmology, Shandong Provincial Hospital, Cheeloo College of Medicine, Shandong University, Jinan, Shandong, China

**Keywords:** autosomal dominant inheritance, congenital cataract, CRYGD, familial segregation, variant

## Abstract

Congenital cataract is an important cause of childhood visual impairment, and genetic factors contribute substantially to its etiology. We report a boy aged 4–5 years with congenital cataract from a family with an apparent autosomal dominant inheritance pattern. Comprehensive ophthalmic examinations were performed in the proband and available relatives. Whole-exome sequencing (WES) in the proband identified several candidate variants, including a heterozygous *CRYGD* c.391T>C (p.Trp131Arg) variant. Subsequent Sanger sequencing was used to validate the candidate variants and assess segregation in available family members. The *CRYGD* c.391T>C (p.Trp131Arg) variant was present in all tested affected family members and absent in the tested unaffected relative, supporting co-segregation with the disease phenotype in this pedigree. Additional variants identified by WES, including *ITM2B* c.537C>G (p.Asn179Lys) and *ASB10* c.1402T>C (p.Ter468GlnextTer6), showed segregation patterns that were less consistent with the familial phenotype. According to ACMG criteria, the *CRYGD* variant remained classified as a variant of uncertain significance. To provide limited biological context, we also reviewed a public gene expression dataset and found lens-enriched expression of *CRYGD*, consistent with its established role in lens biology. This case may provide useful evidence for future variant interpretation and genetic counseling in congenital cataract families carrying *CRYGD* variants; however, further functional studies are required to clarify the pathogenic significance of c.391T>C (p.Trp131Arg).

## Introduction

Congenital cataract is defined as lens opacity present at birth or developing during early infancy. Although congenital cataract is relatively uncommon in the general population, it is an important cause of preventable visual impairment in children worldwide (1). Epidemiological studies indicate that the prevalence of congenital cataract varies across populations, and genetic factors make a substantial contribution to its etiology. In hereditary cases, autosomal dominant inheritance is the most common pattern, although autosomal recessive and X-linked forms have also been reported (2–5).

The molecular mechanisms underlying hereditary cataract are heterogeneous. Mutations in genes encoding lens structural proteins, membrane proteins, cytoskeletal proteins, transcription factors, and other developmental regulators have all been implicated. Among these, crystallin genes are among the most frequently reported disease-associated genes. *CRYGD* encodes γD-crystallin, a major structural protein that is essential for maintaining lens transparency and refractive properties (6, 7). Variants in *CRYGD* have been associated with autosomal dominant congenital cataract in multiple populations (8–11).

Recent studies have further confirmed the marked genetic heterogeneity of congenital cataract. In a 2025 cohort of 19 Chinese families with congenital cataract, WES followed by Sanger-based co-segregation analysis identified likely pathogenic variants in several cataract-associated genes, including *CRYGD, GJA3, CRYAA, CRYBA1, BFSP2, IARS2, ARL2*, and *CRYBB3*, with additional variants classified as VUS. Another 2025 study of 114 probands with congenital cataract also demonstrated broad genotype–phenotype heterogeneity involving isolated cataract, cataract with additional ocular anomalies, and cataract with multisystem abnormalities, supporting the value of WES for variant screening and genotype–phenotype correlation analysis in congenital cataract (12, 13).

Despite substantial progress in identifying disease-related genes, the clinical interpretation of individual rare variants remains challenging, particularly when functional validation is unavailable. Family-based segregation analysis remains an important source of supportive evidence in this setting, especially when whole-exome sequencing identifies multiple candidate variants of uncertain significance.

Similar challenges have been emphasized in recent exome-sequencing studies of pediatric ocular disorders, in which WES improved the identification of candidate molecular diagnoses but also generated rare variants requiring careful interpretation in relation to phenotype, segregation evidence, and functional plausibility (14).

In this study, we describe the clinical and genetic findings of a family with congenital cataract in whom WES identified a *CRYGD* c.391T>C (p.Trp131Arg) variant in the proband. We then performed Sanger sequencing in available family members to assess segregation of this variant within the pedigree. In addition, we briefly reviewed a public dataset to provide supportive biological context for the known lens-associated expression of *CRYGD*. Our aim was to characterize the ocular phenotype of the family, evaluate the segregation pattern of the identified variant, and discuss its relevance in the context of the existing literature.

## Patients and methods

### Compliance with ethical standards

This study was approved by the Medical Ethics Committee of Weifang Eye Hospital, Shandong, China (Approval No. 2024-YNLL-07-01). Written informed consent for treatment and publication was obtained from the patient's legal guardians. All procedures were conducted in accordance with the Declaration of Helsinki. We have de-identified all patient details to protect anonymity.

### Study participants and related exams

The patient and his family in this study were recruited from the Department of Ophthalmology at Weifang Eye Hospital in Shandong Province, China. Comprehensive clinical data were collected from the proband, including visual acuity, intraocular pressure measurement, anterior segment evaluation, vitreous examination, scanning laser ophthalmoscopy (SLO), optical coherence tomography (OCT), ophthalmic B-mode ultrasound, corneal assessment, slit-lamp biomicroscopy, and dilated fundus examination. Available clinical information from relatives was also reviewed.

### Genetic testing and variant interpretation

Whole-exome sequencing (WES) was performed in the proband to identify candidate variants potentially associated with congenital cataract. The sequencing project used in this case was WES015: whole-exome sequencing V6. Candidate variants identified in the proband were interpreted in the context of the clinical phenotype and family history. Subsequently, Sanger sequencing was used to validate candidate variants and assess familial segregation in available relatives, including the proband's father, paternal grandmother, paternal aunt, younger sister, affected male cousin, and unaffected male cousin. The proband's mother was not available for sampling.

Among the candidate variants identified by WES, particular attention was given to the *CRYGD* c.391T>C (p.Trp131Arg) variant because of the known association between *CRYGD* and congenital cataract. Additional candidate variants reported in the proband included *ITM2B* c.537C>G (p.Asn179Lys) and *ASB10* c.1402T>C (p.Ter468GlnextTer6). The *CRYGD* variant is located in exon 3 of transcript NM_006891.4. Variants identified in this study were interpreted according to the 2015 American College of Medical Genetics and Genomics (ACMG) guidelines (15).

### Exploratory public-dataset analysis

To provide limited biological context, we performed an exploratory analysis of the public GSE32334 dataset from the Gene Expression Omnibus database. This analysis was used only to illustrate the lens-associated expression pattern of *CRYGD*. It was not performed on samples from the present family and was not intended as functional validation of the identified variant. Differential expression and visualization were performed using the ggplot2, heatmap, and corrplot packages.

## Results

### Case presentation

The reporting of this study conforms to the CARE guidelines (16). The proband was a boy aged 4–5 years who was admitted to Weifang Eye Hospital, Shandong, China because of decreased vision in both eyes for 5 months. He was the second child of a full-term vaginal delivery and had no history of oxygen therapy or neonatal resuscitation. According to the available medical records and parental report, no seizures, abnormal muscle tone, motor delay, speech delay, cognitive impairment, or other obvious neurological abnormalities were reported in the proband. However, no formal neurodevelopmental assessment was performed; therefore, subtle developmental abnormalities could not be completely excluded. The initial clinical diagnosis was bilateral congenital cataract.

At admission, visual acuity was 0.4 in both eyes. Intraocular pressure was 13.2 mmHg in the right eye and 14.4 mmHg in the left eye. Slit-lamp examination showed clear corneas, normal anterior chamber depth, clear aqueous humor, round pupils with preserved light reflexes, and bilateral lens opacity (C1N2P1). Mild vitreous opacity was noted in both eyes. Fundus examination showed clear optic disc margins, normal disc color, visible peripapillary vessels, leopard-like retinal changes, and visible foveal reflexes in both eyes. Additional ophthalmic examinations, including scanning laser ophthalmoscopy, optical coherence tomography, ocular ultrasonography, biometry, keratometry, corneal topography, and corneal endothelial cell assessment, were also performed.

Under general anesthesia, the patient underwent bilateral cataract extraction with primary intraocular lens implantation, posterior capsulotomy, and anterior vitrectomy during the same admission, with the right eye operated on first, followed by the left eye.

**Figure 1:**
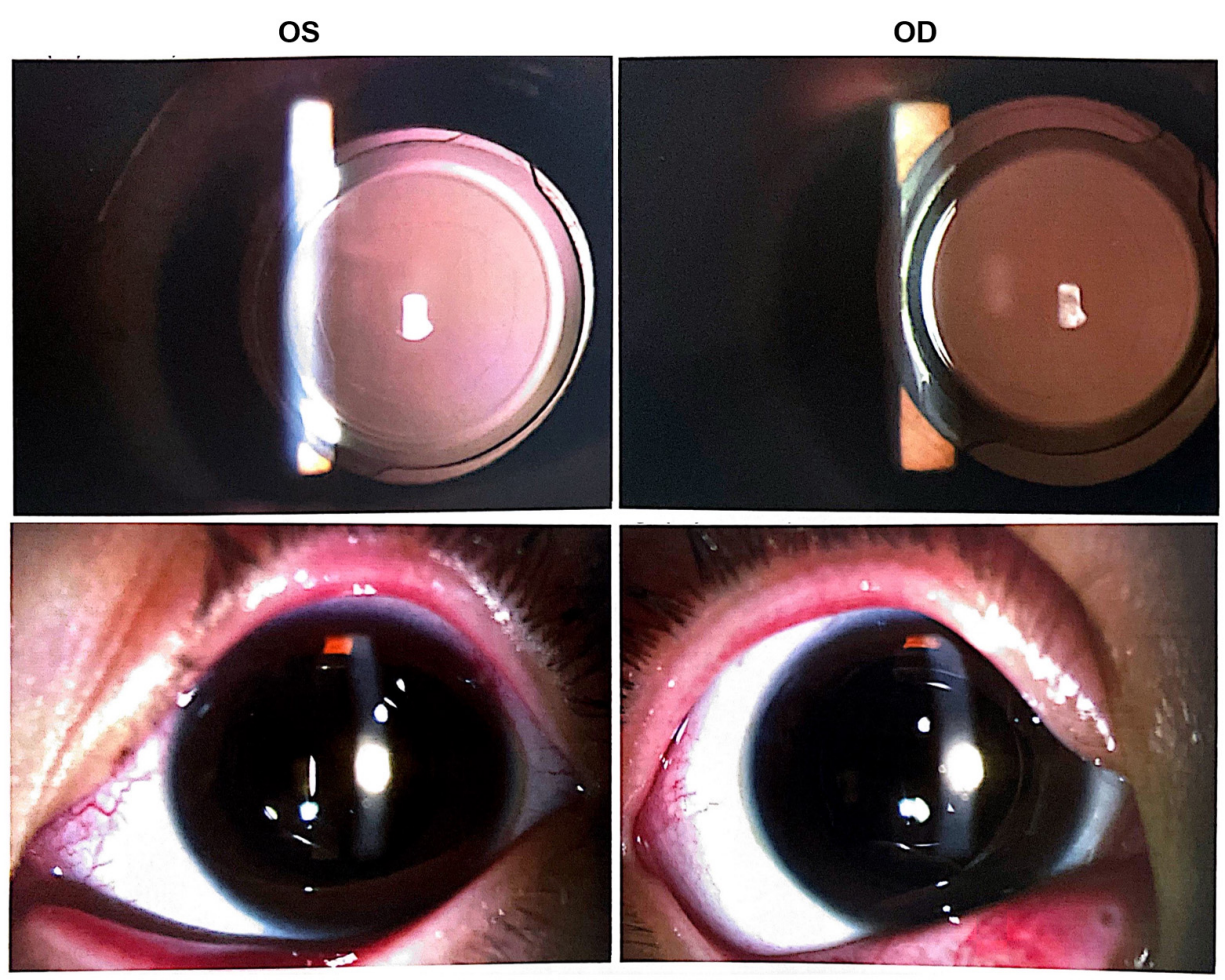
Postoperative anterior segment findings in the proband. Anterior segment photographs obtained during follow-up after bilateral cataract extraction with primary intraocular lens implantation. The images show transparent corneas, a clear visual axis, and well-positioned intraocular lenses in both eyes.

At postoperative follow-up, visual acuity was 0.4 in the right eye and 0.5 in the left eye. Refraction was +0.75 DS/−1.00 DC × 128 in the right eye and −0.50 DS/−2.25 DC × 9 in the left eye. Intraocular pressure was 21 mmHg in both eyes. Anterior segment examination showed transparent corneas, normal anterior chamber depth, mild aqueous flare, round pupils with preserved light reflexes, well-positioned transparent intraocular lenses, and an opened posterior capsule in the visual axis region. Mild vitreous opacity persisted, and the fundus findings were essentially unchanged. Postoperative anterior segment photographs obtained during follow-up showed transparent corneas, a clear visual axis, and well-positioned intraocular lenses in both eyes (Figure 1). No associated systemic abnormalities were identified.

### Family history and clinical findings of relatives

The family history was positive on the paternal side and included multiple affected relatives. According to the available clinical information and family-based testing records, affected individuals included the proband, his father, paternal grandmother, paternal aunt, younger sister, and one male cousin. Another male cousin was clinically unaffected and had no ocular phenotype. The proband's mother was not sampled for genetic testing.

Available ophthalmic records and clinical examinations supported the presence of congenital cataract in the affected family members. Their clinical and genetic results are summarized in Table 1.

**Table 1:** Clinical and genetic findings of the available family members.

Individual	Relationship to proband	Clinical status	Congenital cataract	*CRYGD* c.391T>C genotype	*ITM2B* c.537C>G genotype	*ASB10* c.1402T>C genotype
Proband	Self	Affected	Yes	Het	Het	Het
Father	Father	Affected	Yes	Het	WT	Het
Grandma	Grandma	Affected	Yes	Het	WT	Het
Aunt	Aunt	Affected	Yes	Het	WT	Het
Younger sister	Sister	Affected	Yes	Het	Het	Het
Affected male cousin	Male cousin	Affected	Yes	Het	WT	WT
Unaffected male cousin	Male cousin	Unaffected	No	WT	WT	WT
Mother	Mother	Not sampled	NA	NA	NA	NA

WES was performed in the proband, and Sanger sequencing was used to validate candidate variants in available relatives. The genotype columns indicate the carrier status of each listed variant. Among the candidate variants identified by WES, CRYGD c.391T>C (p.Trp131Arg) showed the strongest consistency with affected status in this family. Het, heterozygous; WT, wild type; NA, not available/not sampled.

### WES findings in the proband

WES in the proband identified several candidate variants of uncertain significance, including *CRYGD* c.391T>C (p.Trp131Arg), *ITM2B* c.537C>G (p.Asn179Lys), and *ASB10* c.1402T>C (p.Ter468GlnextTer6). Among these, *CRYGD* was considered the most clinically relevant candidate gene because of its established association with autosomal dominant congenital cataract.

### Segregation analysis of *CRYGD*

**Figure 2:**
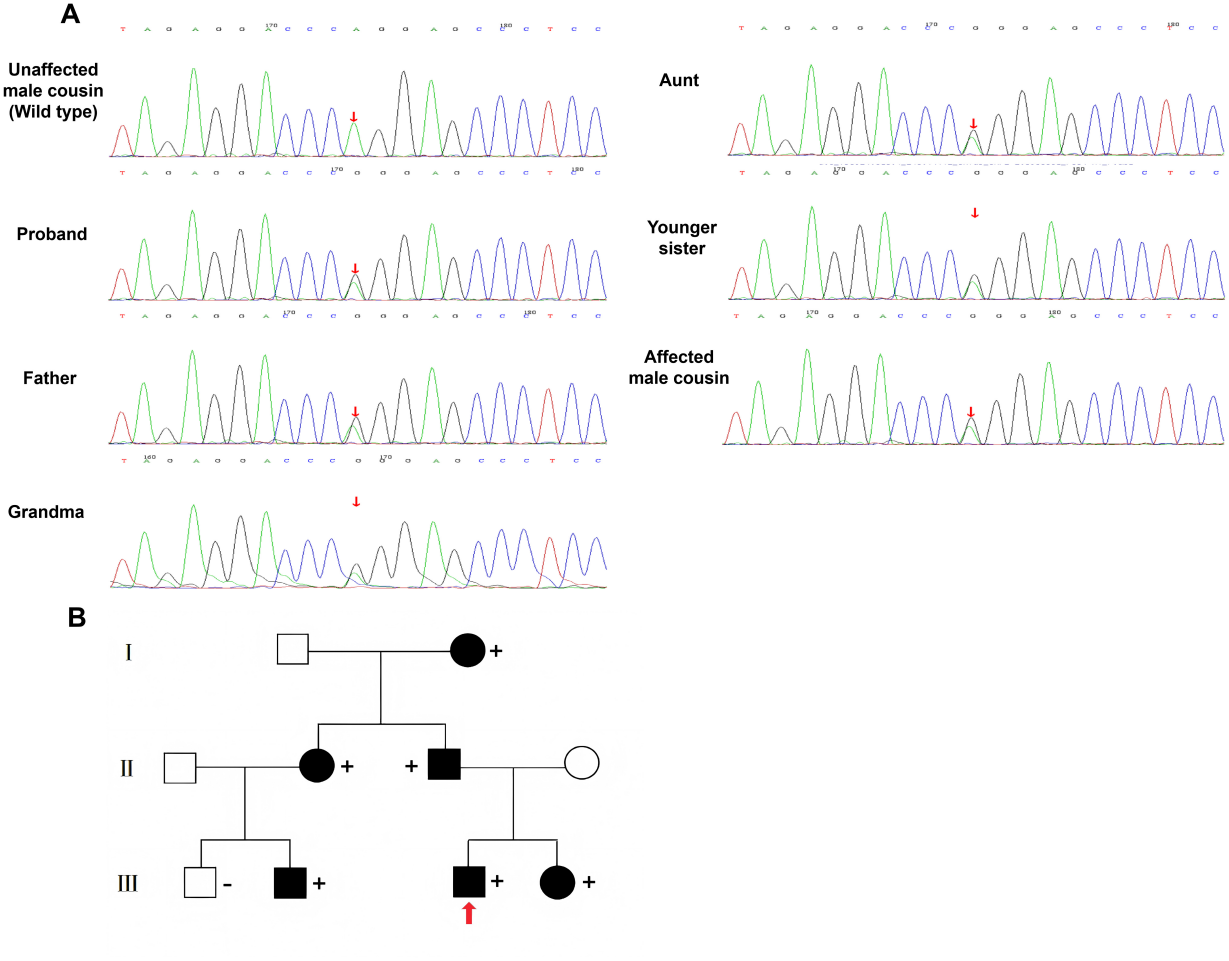
Genetic findings and pedigree of the family. **(A)** Sanger sequencing chromatograms showing the heterozygous *CRYGD* c.391T>C (p.Trp131Arg) variant in the proband. Red arrows indicate the mutation site. **(B)** Pedigree of the family showing segregation of congenital cataract and the *CRYGD* c.391T>C variant. Filled symbols indicate affected individuals, and open symbols indicate unaffected individuals. The arrow indicates the proband. Genotypes for the *CRYGD* c.391T>C variant are indicated as “+” (variant present) or “–” (variant absent).

Sanger sequencing confirmed that the proband carried a heterozygous *CRYGD* c.391T>C (p.Trp131Arg) variant (Figure 2A). The same heterozygous variant was also detected in the proband's father, paternal grandmother, paternal aunt, younger sister, and affected male cousin, whereas the unaffected male cousin did not carry the variant (Figure 2B).

These findings indicate that the *CRYGD* variant was present in all tested affected family members and absent in the tested unaffected relative, supporting co-segregation with the congenital cataract phenotype in this pedigree and a pattern consistent with autosomal dominant inheritance.

### Comparison with other candidate variants

The segregation patterns of the other candidate variants were less consistent with the disease phenotype. The *ITM2B* c.537C>G (p.Asn179Lys) variant was detected in the proband and younger sister, but was absent in the father, paternal grandmother, paternal aunt, affected male cousin, and unaffected male cousin, which did not fit the familial distribution of congenital cataract. Similarly, the *ASB10* c.1402T>C (p.Ter468GlnextTer6) variant was detected in the proband, father, paternal grandmother, paternal aunt, and younger sister, but was absent in the affected male cousin, indicating that it also did not fully co-segregate with the phenotype in this family. Taken together, *CRYGD* c.391T>C (p.Trp131Arg) showed the strongest consistency with affected status among the candidate variants identified by WES.

### Variant classification and computational support

According to ACMG criteria, *CRYGD* c.391T>C (p.Trp131Arg) remained classified as a variant of uncertain significance. Supporting evidence included absence from population databases (PM2_Supporting) and a deleterious computational prediction by REVEL (PP3_Moderate). Therefore, although the segregation findings supported a possible association with the phenotype, the available evidence was insufficient to establish definitive pathogenicity.

### Exploratory public-dataset analysis

**Figure 3:**
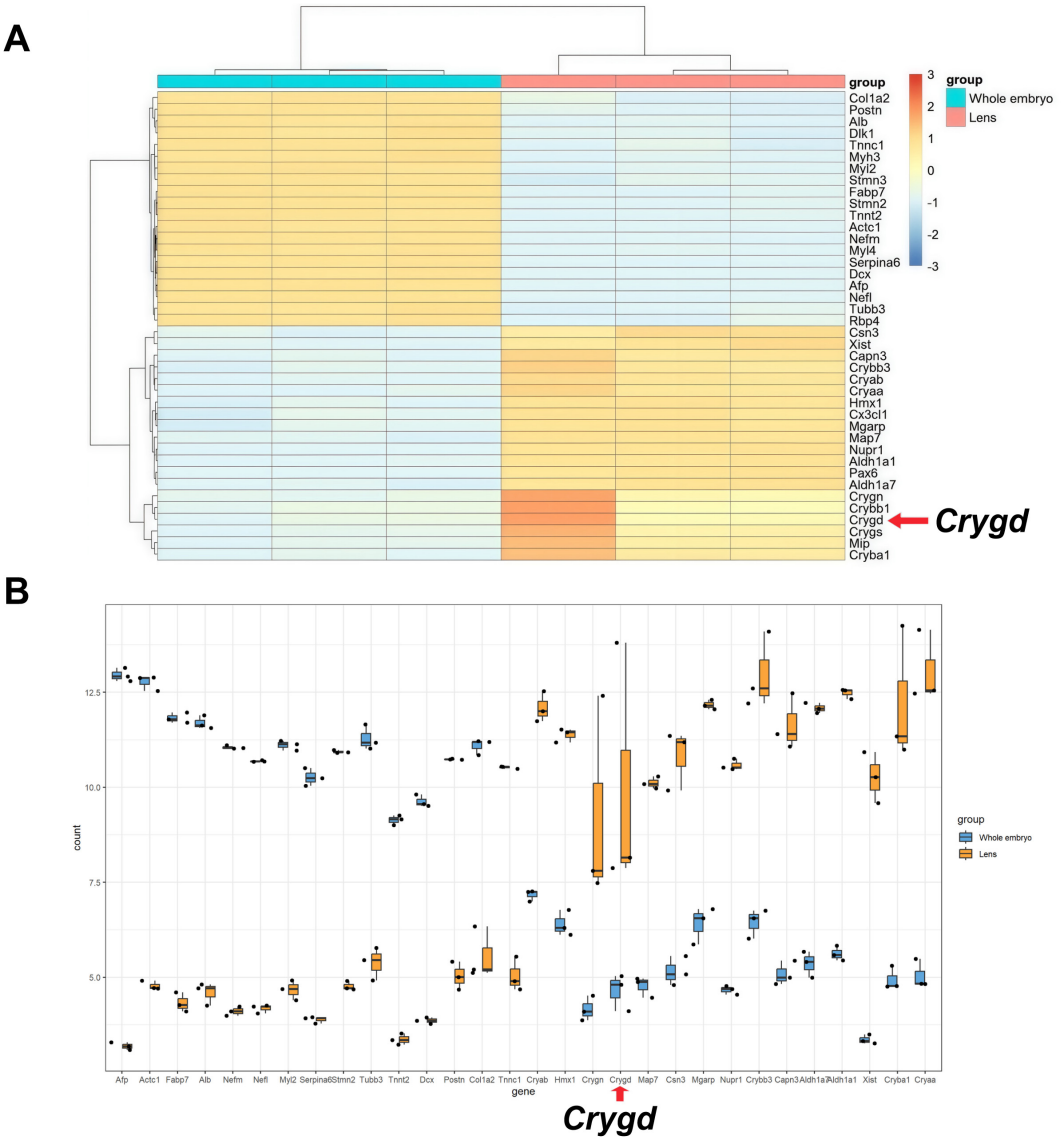
Exploratory public-dataset analysis of *CRYGD* expression in the GSE32334 dataset. **(A)** Heatmap showing the expression pattern of selected lens-related genes in whole-embryo and lens samples. **(B)** Boxplot showing higher expression of *CRYGD* in lens samples than in whole-embryo samples. Differentially expressed genes were defined as those with |log2FC| > 1.0 and *P*-value < 0.05; CRYGD met these criteria and was significantly upregulated in lens samples compared with whole-embryo samples. This analysis was included only to provide biological context and does not represent functional validation of the c.391T>C (p.Trp131Arg) variant identified in this family.

To provide limited biological context, we performed an exploratory analysis of the public GSE32334 dataset. Heatmap visualization showed that lens samples had an expression pattern distinct from that of whole-embryo samples, with higher expression of multiple lens-related genes, including *CRYGD* (Figure 3A). Boxplot analysis further showed that *CRYGD* expression was higher in lens samples than in whole-embryo samples (Figure 3B), which is consistent with its established role in lens biology. Because this analysis was based on a public dataset rather than samples from the present family, these findings were interpreted only as supportive background information and not as functional validation of the c.391T>C (p.Trp131Arg) variant.

## Discussion

In this family with congenital cataract, WES identified a heterozygous *CRYGD* c.391T>C (p.Trp131Arg) variant in the proband, and subsequent Sanger sequencing demonstrated that the variant was present in all tested affected family members and absent in the tested unaffected relative. These findings support co-segregation of the variant with the disease phenotype in this pedigree and are consistent with an autosomal dominant pattern of inheritance.

Congenital cataract is a genetically heterogeneous disorder, and variants in crystallin genes are among the most frequently reported causes of inherited disease. *CRYGD* encodes γD-crystallin, an important structural protein required for lens transparency and refractive stability. Variants in *CRYGD* have been reported in association with autosomal dominant congenital cataract in multiple previous studies (17–19). Recent WES-based studies have also continued to identify cataract-associated variants in Chinese families and broader congenital cataract cohorts, underscoring the usefulness of exome sequencing combined with family-based segregation analysis for inherited cataract (12, 13, 20). Therefore, the present study does not aim to establish a new biological role for *CRYGD* itself; rather, it provides additional clinical and familial evidence relevant to the interpretation of the c.391T>C (p.Trp131Arg) variant in the context of congenital cataract.

An important strength of the present study is that the genetic interpretation is supported not only by identification of a candidate variant in the proband, but also by family-based validation. The candidate variant was identified by WES in the proband and subsequently validated by Sanger sequencing in available relatives, which strengthens the interpretation of the segregation findings.

WES also identified additional variants of uncertain significance, including *ITM2B* c.537C>G and *ASB10* c.1402T>C. However, these variants did not segregate with the phenotype as well as *CRYGD* c.391T>C. The *ITM2B* variant was present only in the proband and younger sister among the tested relatives, whereas the *ASB10* variant was absent in one affected male cousin. Therefore, among the candidate variants identified by WES, the *CRYGD* variant showed the strongest consistency with the familial phenotype and was considered the most relevant candidate variant in this family.

The c.391T>C (p.Trp131Arg) substitution replaces tryptophan with arginine at codon 131. Because tryptophan residues contribute to the structural stability of γD-crystallin, such a substitution may affect protein folding or solubility. In addition, computational prediction supported a potentially deleterious effect. However, the variant remains classified as a variant of uncertain significance according to ACMG criteria, and its pathogenicity cannot be considered established on the basis of the present data alone.

This cautious interpretation is consistent with recent WES-based ocular genetics studies, in which rare candidate variants and VUS were frequently encountered and required integration of population frequency, *in silico* prediction, phenotype consistency, and segregation data (12–14). In the present family, the co-segregation pattern supports *CRYGD* c.391T>C as the most clinically relevant candidate variant, but functional validation and additional unrelated cases will be necessary before definitive pathogenicity can be established.

To provide limited biological context, we also performed an exploratory analysis of the public GSE32334 dataset and found that *CRYGD* showed a lens-enriched expression pattern. This observation is consistent with the known biological role of *CRYGD* as a structural lens protein. However, because this analysis was performed using public data rather than samples from the present family, it should be regarded only as supportive background evidence and not as direct evidence for the functional effect or pathogenicity of the c.391T>C (p.Trp131Arg) variant.

To further contextualize the present family within the broader genetic spectrum of congenital cataract, we summarized representative congenital cataract-associated genes according to typical age of onset, isolated vs. syndromic presentation, key clinical features, inheritance pattern, and implications for genetic testing (Table 2). In addition to crystallin genes such as *CRYAA, CRYBB1, CRYBB2, CRYBA1*, and *CRYGD*, inherited congenital cataract can also involve genes encoding gap junction proteins, membrane proteins, cytoskeletal proteins, and transcriptional or developmental regulators. Representative examples include *GJA3* and *GJA8*, which encode connexins; *MIP*, which encodes a lens membrane protein; *BFSP1* and *BFSP2*, which encode lens intermediate filament proteins; and transcriptional or developmental genes such as *MAF* and *PAX6* (18–21). Syndromic congenital cataract may also involve genes such as *NHS, BCOR*, and *AGK*, which have been reported in congenital cataract with extraocular manifestations, including dental, cardiac, skeletal, metabolic, or developmental features (19, 21). *JAM3*-associated disease has been reported as a severe neonatal syndromic form characterized by hemorrhagic destruction of the brain, subependymal calcifications, and congenital cataracts, also known as HDBSCC (22).

**Table 2:** Representative congenital cataract-associated genes stratified by age of onset and genotype–phenotype features.

Gene	Typical age of onset	Isolated or syndromic presentation	Key associated clinical features	Inheritance	Implications for genetic testing
*CRYGD*	Congenital/early childhood	Usually isolated	Autosomal dominant congenital cataract; variable lens opacity morphology; generally no systemic abnormalities	AD	Prioritize in familial autosomal dominant congenital cataract, especially when segregation supports co-inheritance
*CRYAA*	Congenital/infantile	Usually isolated; occasionally syndromic	Nuclear, lamellar, or variable cataract; may be associated with microcornea in some cases	AD/AR	Consider in early-onset cataract with or without microcornea
*CRYBB1, CRYBB2, CRYBA1*	Congenital/infantile	Usually isolated	Nuclear, lamellar, pulverulent, or variable congenital cataract	AD/AR	Common crystallin candidates in isolated familial cataract
*GJA3, GJA8*	Congenital/infantile	Usually isolated; occasionally with ocular anomalies	Nuclear, lamellar, pulverulent cataract; possible microcornea or other anterior segment features	AD/AR	Consider in familial cataract with variable morphology or microcornea
*MIP*	Congenital/childhood	Usually isolated	Lamellar, cortical, or polymorphic cataract	AD	Consider in autosomal dominant isolated congenital or childhood cataract
*BFSP1, BFSP2*	Congenital/childhood	Usually isolated	Congenital or juvenile cataract with variable lens opacity	AD/AR	Consider when crystallin and connexin genes are negative
*MAF*	Congenital/childhood	Isolated or syndromic	Cataract, anterior segment anomalies; syndromic cases may include developmental delay, seizures, hearing loss, or dysmorphic features	AD	WES is useful when cataract is accompanied by extraocular features
*PAX6*	Congenital/childhood	Ocular developmental/syndromic	Cataract with aniridia, anterior segment dysgenesis, foveal hypoplasia, or other ocular developmental abnormalities	AD	Consider when cataract coexists with anterior segment malformation
*NHS*	Congenital	Syndromic	Nance-Horan syndrome; congenital cataract, dental anomalies, facial dysmorphism, and possible developmental delay	X-linked	Consider in male patients with cataract plus dental/facial features
*BCOR*	Congenital	Syndromic	Oculofaciocardiodental syndrome; cataract with dental, cardiac, facial, and skeletal abnormalities	X-linked	Consider in congenital cataract with multisystem abnormalities
*AGK*	Neonatal/infantile	Syndromic	Sengers syndrome; congenital cataract, hypertrophic cardiomyopathy, skeletal myopathy, lactic acidosis	AR	Consider when cataract is accompanied by cardiomyopathy or myopathy
*JAM3*	Neonatal	Syndromic	HDBSCC: hemorrhagic destruction of the brain, subependymal calcifications, and congenital cataracts	AR	Consider in neonatal cataract with severe neurological involvement or brain imaging abnormalities

This table provides a practical summary of representative congenital cataract-associated genes according to age of onset, isolated vs. syndromic presentation, key clinical features, inheritance pattern, and implications for genetic testing. It is not intended to be an exhaustive list of all cataract-associated genes. The genes and genotype–phenotype features summarized in this table are supported by the cited literature discussed in the accompanying Discussion text. AD, autosomal dominant; AR, autosomal recessive; HDBSCC, hemorrhagic destruction of the brain, subependymal calcifications, and congenital cataracts; WES, whole-exome sequencing.

Based on this genotype–phenotype framework, the phenotype in the present family is more consistent with isolated autosomal dominant congenital cataract than with severe syndromic neonatal cataract. The proband and affected relatives showed congenital cataract without obvious systemic abnormalities. In addition, no seizures, abnormal muscle tone, motor delay, speech delay, cognitive impairment, or other obvious neurological abnormalities were reported in the proband based on available medical records and parental report. This clinical pattern supports *CRYGD* as the most relevant candidate gene among the variants identified by WES, although the c.391T>C (p.Trp131Arg) variant remains classified as a variant of uncertain significance.

The clinical implication of this case lies primarily in family-based variant interpretation and genetic counseling rather than in mechanistic proof. The present report adds segregation evidence from one pedigree that may be useful in future reassessment of the *CRYGD* c.391T>C (p.Trp131Arg) variant as additional cases and functional data become available. However, claims regarding definitive pathogenicity, targeted therapy, or priority inclusion in diagnostic panels would be premature on the basis of the current evidence.

This study has several limitations. First, the evidence is derived from a single family. Second, the proband's mother was not sampled, which limits complete segregation analysis. Third, no functional experiments were performed to directly examine the effect of c.391T>C (p.Trp131Arg) on γD-crystallin. Fourth, detailed phenotypic subclassification was not equally available for all relatives. Finally, although exploratory public-dataset analysis may provide background context, such analysis cannot substitute for family-based or functional validation. Future studies involving additional families, protein functional assays, and independent clinical reports will be needed to clarify the pathogenic significance of this variant.

In conclusion, in this family with congenital cataract, WES identified a *CRYGD* c.391T>C (p.Trp131Arg) variant in the proband, and Sanger sequencing showed that the variant co-segregated with the phenotype in the tested relatives. Among the candidate variants identified by WES, *CRYGD* showed the best segregation with affected status. This case may provide useful evidence for future variant interpretation and genetic counseling, but further functional studies are required before pathogenicity can be confirmed.

## Data Availability

The raw data supporting the conclusions of this article will be made available by the authors, without undue reservation.
